# Assessing the feasibility of measuring residents’ quality of life in English care homes and the construct validity and internal consistency of measures completed by staff proxy: a cross-sectional study

**DOI:** 10.1136/bmjopen-2024-090684

**Published:** 2025-01-29

**Authors:** Ann-Marie Towers, Stacey Rand, Stephen Allan, Lucy Anne Webster, Sinead Palmer, Rachael Carroll, Adam L Gordon, Gizdem Akdur, Nick Smith, Jennifer Burton, Anne Killett, Barbara Hanratty, Julienne Meyer, Karen Spilsbury, Claire Goodman

**Affiliations:** 1 Health and Social Care Workforce Research Unit, King's College London, London, UK; 2 Personal Social Services Research Unit, University of Kent, Canterbury, UK; 3 Centre for Health Services Studies, University of Kent, Canterbury, UK; 4 Academic Unit of Injury, Recovery and Inflammation Sciences, University of Nottingham, Nottingham, UK; 5 Academic Centre for Healthy Ageing (ACHA), Queen Mary University of London, London, UK; 6 Centre for Research in Public health and Community Care (CRIPACC), University of Hertfordshire, Hatfield, UK; 7 School of Cardiovascular and Metabolic Health, University of Glasgow, Glasgow, UK; 8 School of Health Sciences, University of East Anglia, Norwich, UK; 9 Population Health Sciences Institute, University of Newcastle upon Tyne, Newcastle upon Tyne, UK; 10 City University of London, London, UK; 11 National Care Forum, Coventry, UK; 12 School of Healthcare, University of Leeds, Leeds, UK; 13 Centre for Research in Public Health and Community Care, University of Hertfordshire, Hatfield, UK

**Keywords:** Quality of Life, Nursing Homes, Dementia, Psychometrics

## Abstract

**Abstract:**

**Objectives:**

To assess the feasibility of capturing older care home residents’ quality of life (QoL) in digital social care records and the construct validity (hypothesis testing) and internal consistency (Cronbach’s alpha) of four QoL measures.

**Design:**

Cross-sectional data collected in wave 1 of the DACHA (**D**eveloping resources **A**nd minimum dataset for **C**are **H**omes’ **A**doption) study, a mixed-methods pilot of a prototype minimum dataset (MDS).

**Setting:**

Care homes (with or without nursing) registered to provide care for older adults (>65 years) and/or those living with dementia. All homes used a digital record system from one of two suppliers.

**Participants:**

Data were extracted from 748 residents. All permanent residents, aged 65 years or older, were eligible to participate, including those lacking capacity to consent. Temporary residents and residents in their last weeks of life were excluded.

**Outcome measures and analysis:**

The English language versions of Adult Social Care Outcomes Toolkit (ASCOT)-Proxy-Resident, ICEpop CAPability measure for Older people (ICECAP-O), EQ-5D-5L proxy and the QUALIDEM were added to the digital record. As there have not been any previous studies of the structural validity of the English language version of the QUALIDEM, ordinal exploratory factor analysis (EFA) was applied for this measure only. Feasibility (% missing by software provider and measure), % floor/ceiling effects (>15% at lower/upper end of the scales), convergent or divergent construct validity (criterion of >75% of hypotheses accepted) and internal consistency (Cronbach’s alpha ≥0.7) were assessed for all four measures.

**Results:**

The ordinal EFA of QUALIDEM did not replicate the findings of previous research. A six-factor (36 item) solution was proposed and used in all subsequent analyses. There were low rates of missing data (<5%) for all items, except ASCOT-Proxy-Resident Control (5.1%) and Dignity (6.2%) and QUALIDEM item 35 (5.1%). Ceiling effects were observed for the ASCOT-Proxy-Resident and two of the QUALIDEM subscales. None of the scales had floor effects. Cronbach’s alpha indicated adequate internal consistency (α ≥0.70) for the ASCOT-Proxy-Resident, ICECAP-O and EQ-5D-5L proxy. There were issues with two QUALIDEM subscales. Construct validity for all measures was adequate.

**Conclusions:**

The findings support the use of EQ-5D-5L, ASCOT-Proxy-Resident and the ICECAP-O in care homes for older people. The choice of measure will depend on the construct(s) of interest. More research is needed to establish the psychometric properties of the QUALIDEM in an English care home setting.

**STRENGTHS AND LIMITATIONS OF THIS STUDY**

- Quality of life (QoL) measures were added to care home digital social care records (DSCRs) in England and completed by staff proxy.
- Resident views were collected through a single-item QoL question.
- Data was collected about the help residents received to complete the single QoL question.
- Missing demographic data held about residents in DSCRs meant that we were unable to describe or assess the representativeness of residents in the sample.
- The staff were not asked to record whether they completed the measures alone or asked the opinions of residents, family members or colleagues before making their ratings.

## Introduction

 Quality of life (QoL) is an important, person-centred indicator of the quality and effectiveness of long-term social care services and support, including older people living in care homes.^1^,^5^ In England, care homes provide 24-hour personal care and assistance 7 days a week to around 314 577 older people and/or adults requiring dementia care.^6^ Although only 30% of care home facilities in England are registered nursing homes, providing 24-hour medical care from a qualified nurse, they accommodate approximately 50% of the care home population.^7^ This is due to nursing homes having larger capacity than residential care homes and a growing trend for people to receive domiciliary care, meaning by the time they move into a care home they are more likely to have nursing needs.^7^ Despite substantial amounts of data being held about care home residents’ health and care needs, and their use of different parts of the health and social care system, these data are not yet available in an accessible, aggregated form to inform policy, service delivery or user choice.^8 9^ However, the context is changing rapidly in England, with the implementation of a data strategy for health and social care,^10^ aiming to drive digitalisation^11^ and standardise data collected by registered social care providers, with a view to improving interoperability and to facilitate quality care delivery.^12^

The DACHA Study (**D**eveloping resources **A**nd minimum dataset for **C**are **H**omes’ **A**doption)^13 14^ aimed to develop and test a minimum dataset (MDS) for care homes in England. In this context, an MDS is defined as a standardised account of the demographic, social and health characteristics and needs of older people living in long-term care (care home) settings.^13^ Other countries (eg, USA, Canada, New Zealand and regions of the Netherlands and Belgium) have introduced or mandated MDS for care homes.^15^ Equivalent systems have not yet been successfully adapted for the UK context.^8^ Established international instruments, such as the interRAI (formerly known as the Resident Assessment Instrument)^16^ were developed as a crucial tool for assessing and planning care for residents in long-term care facilities, ensuring quality care and compliance with reimbursement requirements. They historically focused on health outcomes. However, there is a growing recognition of the importance of routinely capturing residents’ experiences and well-being,^13 14 17 18^ with QoL measurement now mandatory in residential aged care facilities in Australia^19^ and interRAI users able to purchase QoL surveys for self-report and family proxies.^20^

In the UK, most care homes do not yet capture and summarise residents’ experiences and QoL in a systematic or standardised way.^14^ There is also a lack of consensus around, which constructs that QoL is most relevant for this population.^21^ A multitude of potential instruments measure different QoL constructs, eg, dementia, health-related and social care-related QoL.^2 21^ However, relatively few QoL instruments have been developed and evaluated with specific needs and characteristics of care home residents in mind.^3^ A recent systematic review of QoL instruments used with older adults in care homes found that of 29 instruments identified, only 14 had been psychometrically evaluated with a care home population.^2^ Of these, only two, the Adult Social Care Outcomes Toolkit (ASCOT)^22^ and EuroQoL-5 Dimensions EQ-5D,^23^ had evidence relating to their ability to detect clinically important interventional changes (responsiveness).^2^ The review also stated that no single instrument stood out as best suited to care homes for older people.^2^

Based on this review and scoping of measures available, we identified ASCOT and EQ-5D and three other instruments as potentially appropriate for inclusion in the DACHA MDS: the ICEPop Capability measure for older people (ICECAP-O),^24^ designed for use in economic evaluations; the DEMQOL,^25^ which is a measure of health-related QoL for people living with dementia; and the QUALIDEM,^26^ developed specifically for people with mild to severe dementia and designed for staff completion based on ratings of observable behaviours.^27^ Although ICECAP-O has not yet been psychometrically assessed specifically with care home residents, it has been used with older people, and another systematic review of studies reporting its psychometric properties concluded that it has good construct validity and responsiveness.^28^ DEMQOL-Proxy has been widely used to measure the QoL of people living with dementia, but its psychometric properties are not as well supported as the original self-report measure^25 29 30^ and concerns have been raised about the interpretation of staff proxy responses without an interviewer present.^30^ A new DEMQOL-CH (care home) measure has been developed but requires further development and testing.^30^ Reviews of QUALIDEM indicate the evidence of validity and reliability of the tool, but there is a gap in evidence for the English version.^2 3^

The mode of completion (self-complete/proxy) is critical. In Australia, where QoL measurement is mandated in residential aged care, self-report is the primary assessment method. Data reported for the first quarter of 2024 indicates that 85% of residents were able to self-report via survey (30%) or facilitated interviewsxy-r (55%).^31^ However, in England, response rates to QoL measures among care home residents are very low, with relatively few residents able to self-report,^332^,^34^ highlighting a significant methodological challenge regarding the routine measurement of QoL for this population.

Staff acting as proxies for residents can be controversial when measuring QoL, mostly due to concerns of bias,^35 36^ despite staff frequently collecting data about residents’ physical, psychological and social status to inform assessments and care planning.^37^ Previous research has explored the level of agreement between resident and proxy ratings using different QoL scales, and, in general, the consensus is that agreement is at best ‘fair’.^38 39^ Indeed, staff sometimes worry about judging residents’ ‘subjective state’, both for psychological outcomes, such as QoL,^36 40 41^ and for physical outcomes, such as pain.^33^ Nonetheless, the use of proxy perspectives from care professionals to inform the administration of pain medication, while not considered the ‘gold standard’, is generally accepted.^33^

Ideally, multi-method approaches drawing on observations and adapted qualitative interviews with residents could inform proxy ratings and provide some information about residents’ feelings and experiences.^32 33 38 42^ These methods require time and training to ensure ratings are reliable, with a degree of standardisation between individuals and services.^33^ Previous research has identified a range of barriers to the implementation of tools, such as the lack of time and resources and staff turnover.^43^ Consequently, proxy reporting by staff was chosen for the DACHA study to reflect a need to adopt a feasible and acceptable data collection method that would enable good coverage of data, for all residents (especially in terms of data collection burden for staff and equity for residents without close family or friends). Proxy perspectives are not the same as self-report, they do however offer important insights into the QoL of people who would otherwise be excluded.^41^ They have the potential to be low-burden (staff time) and easy to integrate into routine data collection using digital social care records (DSCRs), both of which are key to the successful implementation of a care home MDS.^43 44^

A detailed description of how the instruments were selected for inclusion in the DACHA MDS is reported elsewhere.^45^ Consultations were conducted with stakeholders, including people working in and with care homes and those with lived experience (eg, family members of residents). Four multi-item measures were chosen to represent the different QoL constructs that stakeholders told us were important to them: health-related (EQ-5D-5L-Proxy), social care-related (ASCOT-Proxy), older people’s capability well-being (ICECAP-O) and dementia-specific (QUALIDEM).^45^ The selection was informed by evidence of psychometric properties,^2^ suitability for proxy completion by staff and consideration of administrative burden (time to complete).^2^ Consultations were particularly important in selecting one dementia-specific QoL measure, with stakeholders choosing QUALIDEM over DEMQOL.^45^ In response to feedback that it was important to give residents an opportunity to rate their own QoL, a single-item QoL measure was also included: the staff were asked to support residents to complete this themselves, where possible.

## Aim

To assess the feasibility of capturing residents’ QoL in DSCRs and assess the construct validity and internal consistency of the four QoL measures, collected by staff proxy.

## Methods and analysis

### Study design

This analysis draws on cross-sectional data from residents’ DSCRs collected in wave 1 of the DACHA study, which was a mixed-methods pilot of a prototype MDS (see study protocol for full details^46^). The study was granted ethical approval from the London Queen’s Square Research Ethics Committee (22/LO/0250).

### Public involvement in the DACHA study

Public involvement (PI) informed the design, conduct and dissemination of the DACHA study. For this study, important public perspectives were taken to be those of people living in care homes, family members of people living in care homes, care workers and care home managers. A family member was a part of the team of people who developed the study and was a co-applicant for the research funding. Additionally, the PI in Research Group (PiRG) at the University of Hertfordshire commented on early versions of the study plan.

Throughout the study, a PI team focused on supporting PI and coordinating involvement with the stages of the project. The PI team was made up of the family member co-applicant, two academic researchers and a director of a care provider advocate organisation.

The involvement of care home residents was facilitated by activity providers based on care homes who met researchers online to co-produce involvement activities that would allow residents to give their opinions and perspectives on key points of the study, including data sharing, priority of different types of data for an MDS and meaning of QoL.^47^ The involvement of family members, care staff and care home managers was facilitated through an online panel, which met quarterly throughout the project. The team consulted the panel on key issues for the project in a timely way to allow the perspectives of the panel members to influence the iterative work of the project. Key issues included priorities for an MDS, the current data environment in care homes, the interpretation of findings of reviews, trusted sources of data, QoL measures, methods for recruitment and support of care homes and their residents and determining audiences for findings from the study and accessible means to communicate key messages.

### Participants

Data were extracted from the DSCRs of 748 older care home residents who consented to the research and were still living in the home at the time of data extraction. The details of resident recruitment are reported as part of the overarching study from which this data was extracted.^48^

Care homes (with or without nursing) were registered to provide care for older adults (>65 years) and/or those living with dementia and were located in one of three participating integrated care systems (ICSs), representing a range of geographic, socioeconomic and organisational contexts.^46^ ICSs are regional partnerships between NHS organisations, local government and others including third sector and social enterprises, which are responsible for coordinating and paying for care in England. As described in the protocol,^46^ all homes were using one of two DSCR systems (referred to as Provider one and Provider two hereafter). Both systems were on NHS Digital’s (now, NHS England) ‘assured solutions list’ for DSCR systems at the time of writing^49^ and had cloud-based systems, which facilitated the extraction of resident-level data for the purposes of populating an MDS. Providers volunteered to participate in the study, and companies did not receive financial reimbursement.

Within participating homes, all permanent residents aged 65 years or older were eligible to take part, including those lacking the capacity to consent. The study was supported by the National Institute for Health and Care Research Clinical Research Network. Residents’ capacity to consent was assessed by a research nurse or member of the research team who visited the care home. Residents with capacity were given a project information sheet, including an ‘easy-to-read’ summary of what to consider and asked to complete a signed consent form. Where residents were not able to consent for themselves, a nominated (ie, professional) or personal (ie, friend or family) consultee was consulted to represent their views and offer advice about participation on their behalf, as required by the Mental Capacity Act in England.^50^ Consultee discussions took place in person, on the telephone or using videoconferencing technology to reduce barriers to engaging with the research. Residents in their last weeks of life (judged by staff) were excluded.

### Measures

Four QoL measures and a single-item QoL rating scale were incorporated into the software of two DSCR providers who had agreed to participate in the study (see study protocol for full details^46^). All measures were in English.

#### QoL

##### Single-item QoL rating scale

A single-item QoL rating scale, taken from the Adult Social Care Survey in England,^51^ was added to software. The question asks respondents to rate their overall QoL, with responses ranging from very good (1) to very bad (7). Where possible, we asked residents to report their own QoL using this item, but where that was not possible, they could receive help or staff could answer on their behalf. To help us interpret responses, we also asked the staff to tick a box indicating the type of help the residents had: no help, someone read the question to them, someone translated the question for them, someone talked through the question with them or someone answered on their behalf (proxy).

##### ASCOT-Proxy-Resident

This is an instrument designed to measure social care-related quality of life (SCRQoL), which forms part of the ASCOT suite of measures.^22 41^ It was developed for proxy completion by unpaid carers or care staff on behalf of adults using social care services, who are unable to self-report.^41^ Proxy respondents are asked to rate eight questions (items) that correspond to ASCOT-Proxy SCRQoL attributes: control over daily life, social participation, occupation (doing things I value and enjoy), personal safety, accommodation comfort and cleanliness, personal comfort and cleanliness, food and drink and dignity. Proxies are not asked to think of a specific time period when responding.

Each attribute was rated according to the proxy’s own opinion (ASCOT-Proxy-Proxy) and the proxy’s view of what they think the person would say (ASCOT-Proxy-Person) against four response statements, which correspond to the ideal state, no needs, some needs and high-level needs. The dual proxy perspectives were designed to reduce any bias associated with the proxy perspective gap, ie, differences in ratings due to proxies spontaneously adopting different approaches to proxy response,^52 53^ as well as specifically for the ASCOT-Proxy, to improve acceptability of the questions to proxy respondents.^41^ Based on these two proxy perspectives, the ASCOT-Proxy provides two measures of SCRQoL, ASCOT-Proxy-Proxy and the ASCOT-Proxy-Person (here called ASCOT-Proxy-Resident).^22^

The ASCOT-Proxy has not previously been used for care home residents as a standalone instrument. However, an adapted version for the proxy report by staff (without the use of dual proxy perspectives) is included in the care homes version (CH4) of ASCOT. This is a feasible, valid and reliable measure, with a higher % completion than the family carer proxy report.^32 33^ A recent study of ASCOT-Proxy completed by family carers of people with dementia, living at home found that of the two measures, only the ASCOT-Proxy-Person/Resident has the same structural characteristics as the original ASCOT self-completion version (SCT4) from which ASCOT-Proxy was adapted.^54^ This finding was replicated in analysis using ASCOT-Proxy data collected in this study, reported elsewhere.^55^ Based on both studies, it was concluded that the ASCOT-Proxy-Proxy perspective is still useful, as it improves the instrument’s acceptability and face validity to proxy respondents, giving them an opportunity to express their own views as well as what they think the person feels.^41^ However, the findings indicate that the ASCOT-Proxy-Resident should be the focus of future analyses, which is why we present only the ASCOT-Proxy-Resident here, with the exception of % missing data (since not reported elsewhere).

As preference weights for ASCOT-Proxy-Resident are not yet available, we applied weights developed for ASCOT-SCT4, ranging from −0.17 (worst possible) to 1 (best possible).^22^

##### ICECAP-O

The ICECAP-O is a measure of capability well-being of older adults developed for use in the economic evaluation of health and social care interventions.^24^ The measure comprises five items that correspond to the following attributes: attachment, security, role, enjoyment and control.^56^ Respondents are asked to indicate which statement best describes their QoL ‘at the moment’. UK preference weights were applied to derive a score from zero (no capability) to one (full capability).^57^ The measure has not been specifically designed or adapted for proxy report. However, it has been applied in the context of older adult care homes as a proxy-report instrument with the recommendation (pending further evidence) that it ought to be completed by professional staff, rather than family members.^58^

##### EQ-5D-5L proxy version 2

The EQ-5D-5L is a five-level version of the EQ-5D, a measure of health-related QoL. The 5 L version was developed from the original three-level (3 L) version to increase reliability and sensitivity, as well as reduce ceiling effects.^23^ It includes the same five dimensions as the EQ-5D-3L, ie, mobility, self-care, usual activities, pain/discomfort and anxiety/depression. The proxy version 2 of the EQ-5D-5L was designed for adults who are not able to self-report due to, ie, cognitive impairment. It asks the (proxy) respondent to rate what they think the person would say (ie, the proxy-person perspective) based on their QoL ‘today’. In this study, the instrument was rated by care staff. Due to concerns raised about the original UK value set for the EQ-5D-5L,^59^ there is an ongoing UK valuation study.^60^ Given this, the recommended mapping function to convert to EQ-5D-3L scores was applied, with UK values applied to generate the index score.^61^

##### QUALIDEM

QUALIDEM is a measure (developed in the Netherlands) of dementia-specific QoL, based on the concept of adaptation to the perceived consequences of dementia: the original Dutch version has been validated and reported in the literature.^26 27^ The questionnaire was translated into English and is available for use,^27^ but psychometric studies have focused on the original Dutch version or translation into German^62^ or Danish.^63^ Our study is the first reported evaluation of the psychometrics of the English translation. The instrument comprises 40 items, which are proxy-reported by care staff on behalf of older adults with mild to severe dementia living in care homes. Of the full list of items, 37 items have previously been found to be scalable onto nine (eight strong and one weak) unidimensional subscales for people with mild to severe dementia.^26 27^ Of these 37 items, 21 are suitable for people with very severe dementia that relate to six of the nine subscales.^26 27^ In this study, all 40 items were included in the care home software system for completion by care staff. Each item is rated on a four-point Likert scale (never to frequently), with indicative items scoring zero for ‘never’ and contra-indicative items scoring three for ‘never’, such that higher scores always indicate better QoL in each subscales. Responses to the items are based on observed behaviour in the previous week. The developers advise against calculating overall scores because subscales differ in content (between two and seven items).^26^

### Cognitive performance

Residents’ cognitive performance was one of the DACHA MDS variables identified as being important but missing from routine data collection in DSCRs.^46^ Cognitive performance was measured using the Minimum Data Set Cognitive Performance Scale (MDSCPS).^64^ The scale consists of five items: dementia diagnosis, short-term memory problems, cognitive skills, ability to communicate and whether or not the person can eat and drink independently. Scores range from 0 (severe impairment) to 6 (intact cognition). The MDSCPS was designed to be completed by care staff on the person’s behalf based on the past week.

### Functional ability

Although care notes within the DSCRs capture residents’ ability to carry out activities of daily living, they were not routinely captured in a standardised and consistent format suitable for quantitative analysis. We therefore added the Barthel index^65^ to the software, which measures the degree of current assistance (at time of assessment) required with ten everyday tasks, including feeding, bathing, grooming, dressing, continence of bowel, continence of bladder, toilet use, transfers (bed to chair and back), mobility of level surfaces and stair negotiation. Items are scored individually (0, unable to do independently; 1, needs assistance; 2, independent) and then summed and multiplied by five, to produce an overall score ranging from 0 (total dependency) to 100 (completely independent).

### Data collection

This study draws on cross-sectional data completed between March and June 2023. The staff completed the measures on behalf of residents, except for the single QoL item, which allowed for self-report (with or without help) or proxy report, depending on residents’ ability. The staff completing the measures varied between homes, ranging from the manager completing them all or named staff being allocated the task. In allocating the work, homes considered workload, consistency of approach and accuracy.^66^ The staff were not given any training to complete the measures, and the only guidance given was that which was already part of the standardised measure (see, Measures, for more information).

Data were extracted by the software providers, in one batch (Provider 1) and four batches (Provider 2) between June and October 2023. Other health and care data pertaining to variables in the DACHA MDS (eg, demographics, delirium, length of stay) were also extracted (see,^48^ for full description). Coded data on residents’ demographics were largely missing from DSCRs in a format suitable for quantitative analysis (despite systems being able to record this) and are therefore not reported here. The completeness of the DSCR data and the feasibility of linking it to other sources of administrative, health and care data for the purposes of populating a care home MDS, is described in full elsewhere.^48^

### Statistical analysis

Complete case analyses were conducted to assess measurement properties, with the sample size for each analysis reported. First, we considered the structural validity^26^ of the forty QUALIDEM items using ordinal exploratory factor analysis (EFA) on polychoric correlation matrices.^67 68^ Ordinal EFA was applied because there have not been previous studies of the structural validity of the English translation, against the original Dutch measure (37 items, nine subscales).^26^ We did not conduct or report EFA for EQ-5D-5L and ICECAP-O, since they are formative measures and EFA/CFA is not appropriate,^69^ nor ASCOT-Proxy-Resident, since EFA and Rasch analysis is reported elsewhere.^29^ For the ordinal EFA with QUALIDEM items, we applied Horn’s parallel analysis, using principal component analysis, without rotation, to estimate randomly generated eigenvalues in 5000 random correlation matrixes, using the 95th percentile.^70^,^73^ Factors were retained when the observed exceeded the random principal component eigenvalues.^70 74^ When two or more factors were retained, promax rotation was applied. Items were taken to load onto a factor if the factor loading (rotated for ≥2 factors) was ≥0.40.^75^

Descriptive statistics were reported for all measures (informed by the EFA for QUALIDEM), alongside indicators of data completeness. Complete missingness (% missingness, due to non-completion of all items in measure) and partial missingness (% missing, due to partial completion) were reported by the QoL measure. The % missing (by item) was also reported, in full, for QoL measures, where one or more items have ≥5% missing data. Missingness is reported separately by software provider because the two systems handled missing data in different ways (Provider 1 forced completion and Provider 2 did not). In both cases, there are issues with using % complete or partial missingness as indicators of feasibility, which relate to each system’s functionality, and need to be considered in data interpretation. Specifically, Provider 1’s system required forced completion for items in ICECAP-O, QUALIDEM and EQ-5D-5L; for ASCOT-Proxy only, it was possible to select ‘don’t know’, coded as missing data. For Provider 2, the system did not require completion of all items. There was no user prompt if items were not completed. Therefore, it was possible to only partially complete each measure, due to either deliberate non-completion (ie, due to item acceptability or feasibility) or user error of omission. Due to these limitations, <5% missingness by the item was applied as the primary indicator of feasibility.

The floor (lowest score) and ceiling (highest score) percentages were also considered for each measure, with a floor or ceiling effect indicated if reported by ≥15% of respondents.^76^ For QUALIDEM, we report descriptives, completeness, floor and ceiling only for those residents rated as having ‘borderline’ to ‘severe’ cognitive impairment on the MDS CPS^64^ because only six of the nine original QUALIDEM subscales are recommended for people with ‘very severe impairment’.^26^ Since there were only n=79 residents rated ‘very severe’ on the MDS CPS, we were unable to run the analysis for these cases separately.

Construct (convergent or divergent) validity of the QoL measures was assessed by hypothesis testing about expected relationships with other outcome measures, using Spearman rank correlation (p value less than 0.01). Correlation coefficients were interpreted as weak (<0.3), moderate (0.3 to 0.5) or strong (>0.5).^77^ These hypotheses were based on previous studies using the ASCOT-Proxy or other ASCOT measures (SCT4, CH4) or developed *a priori* based on the measurement constructs (see table 5). A criterion of >75% of hypotheses accepted was considered as sufficient evidence of construct validity.^78^

Internal consistency was considered using Cronbach’s alpha, with a value of ≥0.7 taken to be acceptable.^79^ COSMIN reporting guidance advises that an assessment of internal consistency is not required for formative measures.^69^ Preference-based measures (EQ-5D-5L, ASCOT and ICECAP) are generally accepted to be formative^80^; however, for comparability with previous research,^28 32 38^ we have assessed internal consistency in this study.

We used the COSMIN Study Design Checklist rule of thumb for the adequacy of the sample size for EFA, internal consistency and construct validity by hypothesis testing. In all cases, >100 participants responded ‘very good’.^69^

All analyses were conducted in STATA 16.^81^

## Results

### Structural validity of QUALIDEM

This is reported first because the findings inform other analyses and reporting. The ordinal EFA of QUALIDEM did not replicate the nine-factor structure proposed by the original developers, ie, 37 items relating to nine subscales of dementia-related QoL.^26 27^ First, we had to omit two items (33, criticises the daily routine and 37, indicates feeling worthless) due to linear dependencies that led to indefinite matrices when conducting ordinal EFA. With the remaining 38 items, Horn’s parallel analysis indicated a six-factor solution, for which 36 items loaded onto at least one of the six factors with loading of ≥0.40 (see table 1). Where items loaded onto more than one factor, they were attributed to the highest loading, and the secondary loading is reported in brackets. The six factors (36 items) related to positive and negative affect (including mood and behaviour) (Subscale 1. 15 items), restlessness, tension and agitation (Subscale 2. 5 items), enjoyment of meals/food (Subscale 3. 2 items), boredom and disengagement (Subscale 4. 6 items), social engagement (Subscale 5. 5 items) and anxiety or low mood (Subscale 6. 3 items). Items 17 and 26 did not load on to any of these six factors. This six-subscale (36 item) solution is used in all subsequent analyses.

**Table 1:** Exploratory factor analysis of QUALIDEM (n=540)

	Factor one loadings	Factor two loadings	Factor three loadings	Factor four loadings	Factor five loadings	Factor six loadings	Uniqueness
Is cheerful	0.93						0.19
Makes restless movements		0.93					0.30
Has contact with other residents	(.51)				0.54		0.35
Rejects help from nursing assistants	0.53						0.21
Radiates satisfaction	0.77						0.44
Makes an anxious impression						0.73	0.30
Is angry	0.47	(.45)					0.25
Is capable of enjoying things in daily life	0.65						0.27
Does not want to eat			0.93				0.15
Is in a good mood	0.91						0.19
Is sad						0.70	0.27
Responds positively when approached	0.81						0.15
Indicates that he or she is bored				0.60			0.52
Has conflicts with nursing assistants	0.52						0.18
Enjoys meals			0.91				0.15
Is rejected by other residents		0.67					0.41
Accuses others							0.38
Takes care of other residents					0.87		0.25
Is restless		0.98					0.19
Openly rejects contact with others	0.43						0.33
Has a smile around the mouth	0.88						0.24
Has tense body language		0.51					0.45
Cries						0.50	0.48
Appreciates help he or she receives	0.71						0.17
Cuts himself/herself off from environment	(.42)			0.59			0.40
Finds things to do without help from others							**0.60**
Indicates he or she would like more help				0.83			0.40
Indicates feeling locked up				0.41			0.41
Is on friendly terms with one or more residents					0.61		0.24
Likes to lie down				0.48	(.42)		0.59
Accepts help	0.57						0.30
Calls out					0.40		0.42
Criticises the daily routine (omitted)							Omitted
Feels at ease in the company of others	0.67						0.41
Indicates not being able to do anything				0.66			0.40
Feels at home on the ward	0.74						0.46
Indicates feeling worthless (omitted)							Omitted
Enjoys helping with chores on the ward					0.51		**0.71**
Wants to get off the ward		0.44					0.41
Moods can be influenced in positive sense	0.74						0.41

Only factors loading of ≥0.40 are reported. Where items loaded onto more thatn one factor, the secondary factor loading is reported in brackets. Items with uniqueness of ≥0.60 are shown in **bold**.

### Feasibility

Missing data are reported in table 2. Missing data (% partial and complete missingness) were higher for Provider 2, compared with Provider 1. Differences in % partial missingness may be due to differences between the two software systems—specifically, Provider 1 required forced completion, but Provider 2 did not. Differences in % of complete missingness may have been affected by the longer period between consent and data completion for Provider 2, due to delays in finalising and releasing the instruments to care homes, and participants no longer being resident (ie, due to hospitalisation or death). The DSCR data used in this analysis (unlike the full linked MDS^48^) was limited insofar as we were unable to identify and exclude these residents.

**Table 2:** % missing data

	Complete missingness % of sampleProvider 1*	Complete missingness % of sampleProvider 2*	Partial missingness % of sampleProvider 1*	Partial missingness % of sampleProvider 2*	% missing data<5% for all items, where the measure is partially completed?
ASCOT-Proxy-Proxy	7.1%	21.3%	4.1%	9.2%	**No** †
ASCOT-Proxy-Resident	9.4%	24.2%	5.9%	13.7%	**No** †
ICECAP-O	8.8%	25.4%	None	<2%	Yes
EQ-5D-5L Proxy 2	7.6%	12.3%	<2%	2.4%	Yes
QUALIDEM 1: Positive or negative affect	11.2%	13.7%	80.4%	9.1%	**No** †
QUALIDEM 2: Restlessness, tension and agitation	11.2%	13.7%	<2%	4.9%	Yes
QUALIDEM 3: Enjoys meals/food	11.2%	14.4%	<2%	6.3%	Yes
QUALIDEM 4: Boredom and disengagement	11.2%	13.7%	4.2%	12.9%	**No** †
QUALIDEM 5: Social engagement	11.2%	13.7%	<2%	5.1%	Yes
QUALIDEM 6: Anxiety and low mood	11.2%	13.7%	<2%	3.2%	Yes
ASCS Overall QoL (single item)	8.2%	20.9%	n/a	n/a	n/a

For QUALIDEM only, we excluded residents with very severe dementia on the Minimum Data Set Cognitive Performance Scale (MDS CPS) (n=79) to leave an overall sample of *n*n=669 split between provider 1 n=143 and provider 2 n=526.

Bold text indicates that the criterion for psychometric evaluation was not met.

* Overall sample *n*n=748, Provider 1 *n*n=170, Provider 2 *n*n=578.

† Full details reported in table 3.

‡ Due to missing item in the software provider system.

Due to these data limitations, it is difficult to interpret the meaning of % partial and complete missingness. As such, the feasibility of care staff completing the QoL instruments on behalf of residents was assessed by examining % missing data by item when at least one item in a measure had been completed (see table 2, final column, and table 3). Apart from QUALIDEM item 1, which was omitted in the first release of the software to care homes by Provider 1, none of the QoL items had % missing data of ≥7%. There were low rates of missing data (<5%) for all items in the four QoL instruments, except ASCOT-Proxy-Resident Control (5.1%) and Dignity (6.2%) and QUALIDEM item 35 (5.1%) Overall, this indicates that the QoL instruments were feasible for care home staff to complete.

**Table 3:** Missing data by item

Measure	% Missing
ASCOT-Proxy-Proxy (n=613)	
Food and drink	None
Home comfort and clean	<2%
Personal comfort and clean	<2%
Social participation	<2%
Occupation	<2%
Control over daily life	<2%
Personal safety	<2%
Dignity	**6.2%**
ASCOT-Proxy-Resident (n=592)	
Food and drink	3.4%
Home comfort and clean	3.4%
Personal comfort and clean	2.2%
Social participation	3.9%
Occupation	4.9%
Control over daily life	**5.1%**
Personal safety	3.2%
Dignity	**6.8%**
QUALIDEM 1: positive or negative affect (n=574)	
Is cheerful	19.9%*
… All other items	<2%
QUALIDEM 4: boredom and disengagement (n=574)	
Indicates he or she is bored	3.3%
Cuts him/herself off of environment	<2%
Indicates he or she would like more help	2.6%
Indicates feeling locked up	3.5%
Likes to lie down	2.1%
Indicates not being able to do anything	**5.1%**

* Due to omission of item in the first release of the measure in software pProvider 1’s system. If those cases affected by this error are not considered, the % missing is <2%.

Of the n=613 cases (81%) where the single-item ASCS QoL item was completed, 14.9% (n=91) was completed by the resident without help and 27.8% (n=170) was completed by staff proxy, without any involvement of the resident. The remaining responses, except one case of missing data (57.3%, n=351), were completed by the resident with assistance from care staff, for example, to read, talk through and/or translate questions.

### Floor and ceiling effects

Descriptive statistics and summary of the psychometrics, including % floor/ceiling, are reported in table 4. Social care-related QoL, measured by the ASCOT-Proxy-Resident, was higher than expected with a mean of 0.83 and a ceiling effect of >15% at the upper end of the scale. There were no floor or ceiling effects for the ICECAP-O, measuring capability well-being. Two of the QUALIDEM subscales had ceiling effects (2, 3) with >15% at the upper end of the scale. The staff used the full scale to capture residents’ health-related QoL using the EQ-5D-5L proxy with less than 2% of scores at the top and bottom of the range. There was a mean score of 0.33, which is in line with previous research.^32^

**Table 4:** Descriptive statistics and internal consistency

	Mean, Std. Dev	Range	N*	% floor	% ceiling	Cronbach’s α (no. of items)
ASCOT-Proxy-Resident †	0.83, .019	−0.17 to 1.00	503	<2%	**17.7%**	0.83 (8)
ICECAP-O	0.73, 0.21	0 to 1	583	<2%	3.4%	0.81 (5)
EQ-5D-5L Proxy 2	0.33, 0.35	−0.59 to 1	650	<2%	<2%	0.74 (5)
QUALIDEM 1: positive or negative affect	35.52, 7.50	11 to 45	418	0%	7.4%	0.92 (15)
QUALIDEM 2: restlessness, tension and agitation	10.87, 3.42	1 to 15	553	0%	**19.7%**	0.78 (5)
QUALIDEM 3: enjoys meals/food	4.65, 1.37	0 to 6	542	<2%	**38.8%**	0.72 (2)
QUALIDEM 4: Bboredom and disengagement	12.24, 3.58	1 to 18	507	0%	6.7%	0.**66 (6**)
QUALIDEM 5: social engagement	7.78, 3.44	0 to 15	552	<2%	<2%	0.72 (5)
QUALIDEM 6: anxiety and low mood	5.65, 2.12	0 to 9	562	<2%	9.3%	0.75 (3)
ASCS overall QoL item from best (1) to worst (7)	3.16, 1.08	1 to 7	613	n/a	n/a	n/a
Barthel index from lowest (0) to highest (100) independence	41.49, 30.19	0 to 100	630	n/a	n/a	n/a
MDS CPS from very severe impairment (0) to intact (6)	3.10, 2.01	0 to 6	582	n/a	n/a	n/a

Bold text indicates that the criterion for psychometric evaluation was not met.

For QUALIDEM only, we excluded residents with very severe dementia on the Minimum Data Set Cognitive Performance Scale (MDS CPS) (n=79) to leave an overall sample of *n*n=669 split between pProvider 1 (n=143) and pProvider 2 (n=526).

* Overall sample *n*n=748, Provider 1 *n*n=170, Provider 2 *n*n=578.

† The descriptive statistics and psychometrics are only reported further for the ASCOT-Proxy-Resident, using preference weights developed for the ASCOT-SCT4.^22^ For further discussion and justification of our focus on ASCOT-Proxy-Resident, not –Proxy-Proxy, see.^54 55^

The mean Barthel and cognitive performance scores were as expected for this population based on previous research, indicating severe dependency.^33 82^ Although we do not have demographic information, these are reassuring indicators of the representativeness of the sample to the care home population of each ICS.^39^

### Internal consistency

Cronbach’s alpha indicated adequate internal consistency (α ≥0.70) for the ASCOT-Proxy-Resident, ICECAP-O, EQ-5D-5L proxy and QUALIDEM, except for QUALIDEM Subscale 4 (boredom and disengagement) based on the EFA conducted for this study (α ≤0.70, table 1). QUALIDEM Subscale 1 (positive and negative affect) also had very high internal consistency (α ≥0.90), which may indicate redundancy of items.

### Construct validity

The construct validity analysis by hypothesis testing is reported in table 5. Despite being completed by staff proxies, the expected associations between the different QoL measures were generally borne out. As >75% of the proposed hypotheses were accepted for each set of hypotheses, there is evidence of adequate construct validity for all four measures.

**Table 5:** Construct validity by hypothesis testing

	Hypotheses	Spearman rank correlation (N)	Hypothesis accepted?
ASCOT-Proxy-Resident	**Strong positive association**:ICECAP-O—based on previous research which found a strong positive association between ASCOT-SCT4 and ICECAP-O for older adults receiving social care.^91^	.60** (441)	Yes
	**Strong negative association:**ASCS Overall QoL Item (negatively scored)—based on previous research using ASCOT, which has found strong associations with overall QoL.^91^	−0.54** (497)	Yes
	**Moderate positive association**:EQ-5D-5L Proxy 2—based on previous research with care home residents, which found moderate positive associations when using the ASCOT-CH4 (mixed-methods) toolki.t^32^	.32** (488)	Yes
	QUALIDEM subscales based on EFA reported in this study—based on conceptual similarities with ASCOT items, particularly Food and drink, Social participation, Occupation (meaningful activity), Control over daily fife and known associations between ASCOT and overall QoL scales in previous research.^91^		
	QUALIDEM 1: positive and negative affect*	.49** (309)	Yes
	QUALIDEM 2: restlessness, tension and agitation*	.49** (426)	Yes
	QUALIDEM 3: enjoys meals/food*	.33** (418)	Yes
	QUALIDEM 4: boredom and disengagement*	.41** (398)	Yes
	QUALIDEM 5: social engagement*	.39** (426)	Yes
	QUALIDEM 6: anxiety and low mood*	.37** (435)	Yes
	Barthel index of independence—based on previous research with care home residents, which found moderate positive associations when using the ASCOT-CH4 (mixed-methods) toolkit.^32^	.34** (456)	Yes
	MDS cognitive performance scale— based on previous research with care home residents, which found moderate positive associations when using the ASCOT-CH4 (mixed-methods) toolkit.^26^	.45** (468)	Yes
ICECAP-O	*See above for ASCOT-Proxy-Resident*		
	**Strong negative association:**ASCS Overall QoL Item (negatively scored)—based on previous international research involving older adults, which has found moderate to strong associations between the ICECAP-O and other measures of self-reported QoL.^28^	−0.53** (527)	Yes
	**Moderate to strong positive association:**EQ-5D-5L Proxy—based on previous international research involving older adults, which has found moderate to strong associations with the EQ-5D measures.^28^	.60** (565)	Yes
	QUALIDEM 5: social engagement*— based on conceptual similarities between the items in this subscale and items in the ICECAP-O (ie, attachment, enjoyment).	.40** (485)	Yes
	Barthel index of independence—based on previous research, which has found strong positive associations between the ICECAP-O and the Barthel.^92^	.55** (510)	Yes
	MDS cognitive performance scale—based on previous international research involving older adults, which has found moderate to strong associations with the cognitive functioning.^28^	.49** (549)	Yes
EQ-5D-5L Proxy 2	*See above for ASCOT-Proxy and ICECAP-O*		
	**Moderate negative association:**ASCS Overall QoL Item (negatively scored)—based on the hypothesis that self-rated QoL will be associated with health-related QoL (measured by EQ-5D-5L) but not strongly because residents are receiving care to compensate for the impact of their health and care needs on their QoL.	−0.28** (592)	**No**
	**Moderate positive associations:**MDS cognitive performance scale—based on previous research conducted with care home residents in England indicating many residents have impaired physical and cognitive functioning.^33^	.40** (612)	Yes
	**Strong positive associations:**Barthel index of independence—based on previous research conducted with care home residents in England^33^ and because both scales assess residents’ functional ability.	.84** (565)	Yes
QUALIDEM	*See above for ASCOT-Proxy-Resident and ICECAP-O and EQ-5D-5L Proxy 2*		

* Only including respondents, whose MDS CPS score was not ‘very severely impaired’.

† p<.01, **p**p<0.001.

EFAexploratory factor analysisQoLquality of life

The ASCOT-Proxy-Resident and ICECAP-O were strongly associated with one another and the overall QoL single item, which is as expected given the shared underlying construct of QoL. They also had the expected associations with conceptually similar subscales of the QUALIDEM reported in the EFA of this study. The EQ-5D-Proxy 2, however, has a moderate association with these other measures, and a much stronger association with the Barthel, which is an index of independence. This reflects the health-related focus of the EQ-5D-5L, which may also explain why we only found a weak association between the EQ-5D-5L Proxy 2 and the ASCS overall Qol item—residents are receiving care to compensate for the impact of their health and care needs on their QoL.

## Discussion

This study sought to explore the feasibility of routinely capturing QoL data about care home residents and assessed the construct validity and internal consistency of four QoL measures, completed by staff proxies. The measures were integrated into two DSCR systems, both of which were on the NHS Digital ‘assured solutions’ list, yet the two systems differed in their tolerance of missing data and how they implemented the measures in participating homes. Forced completion of the items within the measures led to fewer missing data overall. Delays finalising and releasing the instruments to care homes for Provider 2 led to a longer gap between resident recruitment and completion of the measures and reduced the time staff had to complete the measures before data extraction. This may also have contributed to higher rates of non-completion (% complete missingness), due to participants no longer being resident in the care home (ie, due to hospitalisation or death), although we cannot verify this from the data extracted here.

Implementation issues aside, once the staff began to complete the QoL measures they were likely to finish them, indicating completion by staff proxy is a feasible method of collecting QoL data for the purposes of a care home MDS. Only the ASCOT-Proxy-Resident Dignity item had more than 6% of missing data. This item is important when capturing the impact of social care on people’s QoL^22^ and was acceptable during the development of the ASCOT-Proxy,^41^ yet staff and family proxies alike appear to find this more difficult to judge than the other domains.^41 54^ ASCOT-Proxy-Resident Dignity asks the proxy to rate the effects of help from paid carers on how the resident thinks and feels about themselves (from the resident’s perspective). This involves several empathetic perspective shifts, which proxies may find difficult to navigate cognitively and/or judge through their day-to-day interactions with the person. Qualitative interviews and focus groups with the staff exploring their experiences of completing the measures have been reported separately.^66^

Previous reviews have proposed the QUALIDEM as among the best QoL measures for the use in data collection in care homes for older people.^3 4^ It was the dementia QoL scale that achieved the most support from stakeholders for the DACHA study, hence its inclusion in the study.^45^ However, the mixed nature of prior evidence of its psychometric properties has been noted.^4^ The developers of the original measure, in Dutch, indicated some issues with scalability and internal reliability, for some subscales; furthermore, the assessment of the structural validity of the German translation did not support the original subscales.^62 83^ Here, we present the first EFA on the English translation, which indicated a six-factor solution, using 36 of the original 40 items. These do not correspond to the original Dutch, which is recommended by the developers for scoring of the items into subscales for the English translation or the German translation subscales.^27 83^ There were also issues with the internal consistency for two of the subscales: boredom and disengagement (subscale 4), which did not meet the criteria (α ≤0.70, table 1) and positive and negative affect (subscale 1), which had very high internal consistency (α ≥0.90), potentially indicating redundancy of items. Despite the adequate construct validity, the mixed evidence for internal consistency and structural validity of translated versions, both in this study of the English language version and previous studies of other translated versions, means that we are not able to recommend the inclusion of QUALIDEM in a UK care home MDS at this time. Future research should establish the replicability of these findings with the English translation and consider the implications for validity.

Overall, the psychometric evidence (internal consistency, construct validity and also, structural validity, where appropriate) supported the use of the other three multi-item measures. These were also the measures that had the best psychometric evidence when considering the measures to include in the DACHA MDS, as well as the alignment to the constructs of (social/long-term) care-related and health-related QoL that are most useful in reflecting on the quality and effectiveness of care delivered in the care home context.^2^ Of these, only the ASCOT-Proxy-Resident had a ceiling effect. This is common for ASCOT and reflects the fact that ASCOT captures the impact of social care on QoL—if good quality care is being delivered and meeting people’s needs and preferences, they will score highly, and this is a desirable state. This is supported by the findings of previous research in care homes showing a positive association between residents’ SCRQoL and care home quality ratings^33 84 85^ and by analysis using linked health and social care data from this study, which replicated these findings for ASCOT-Proxy Resident but did not find an association between care quality and outcomes for EQ-5D or ICECAP-O.^86^

We found a higher than expected mean score for residents’ SCRQoL, using the ASCOT-Proxy-Resident, compared with previous research (0.83 vs 0.74-0.77).^33^ Previous studies used the mixed-methods tool (ASCOT-CH4), in which *trained* researchers rated residents’ SCRQoL after conducting structured observations, staff interviews and speaking to residents.^32 33^ In the DACHA study, the staff were not given any training before completing the measures, they were only provided with limited support and the guidance included by the measure’s developers at the start of each scale. It is possible that the staff rated residents’ SCRQoL more highly because they felt that low ratings would reflect poorly on the quality of care being provided. However, qualitative work, reported elsewhere^66^ indicated that staff completed the measures with integrity, seeking to understand residents’ perspectives when completing measures on their behalf. Nonetheless, there was evidence of mistrust from care home staff who completed the measures about how this data would be used and for what purpose.^66^ An ongoing international work using ASCOT with care providers indicates that assimilating QoL measures into everyday practice through care planning reframes outcome measurement as part of care delivery and an ongoing commitment to quality improvement,^87 88^ rather than an auditable metric to which care homes might be held accountable.

For ICECAP-O, we cannot compare mean scores with previous research because the measure has not previously been used in UK care homes. However, the DACHA sample had a lower proxy-reported mean QoL score compared with a community sample of older people (>65 years) in England (0.73 vs 0.81), which is consistent with differences in the functional ability of the two samples.^89^ This provides tentative support to the qualitative evidence that staff completed these additional measures with integrity^66^ and tried to respond from the position of the resident themselves (the proxy-resident perspective), rather than giving their own view (the proxy-proxy perspective). However, a limitation is that the ICECAP-O was not designed for completion by proxies and therefore unlike the ASCOT-Proxy-Resident or EQ-5D-5L-Proxy 2, we cannot be sure which perspective staff adopted or whether there was variation between and within care homes in how staff interpreted the task. Most residents (57.3%) required help (eg, to read or talk through the question) to complete the single-item QoL scale, with over a quarter completed by staff proxy with no resident input at all. Only 15% of residents in this study completed this question without any help at all. This is in line with previous research in English care homes for older adults, which found that less than 25% of residents could give their views of their own care-related QoL using a structured ASCOT questionnaire, whereas around 60% could talk about the care-related QoL if questions were asked in a flexible, qualitative interview.^33^ It is likely that if residents had self-completed the longer QoL measures in DACHA, we would have had substantial missing data, affecting the ability to generate overall scores and interpret the results.

A limitation of this study is that, despite expecting staff to complete the measures by proxy, we cannot be sure of the extent to which staff discussed the questions with residents before/while completing them.^66^ We only collected this information for the single-item QoL question. As outlined above, one of our shortlisted measures (the ICECAP-O) has also not been designed specifically with proxy reporting in mind and so caution is advised around how this measure is interpreted. Ideally, in future data collection, detail on exactly how proxies completed the standardised measures (ie, on their own, after speaking with the resident, or by asking the resident to give their own view) should be captured and considered in analyses. Given the evidence presented here supporting the inclusion of ICECAP-O in a care home MDS, future work to develop and validate a proxy-report version would be welcome.

Another limitation of this study is that most care homes did not complete the demographic fields in the DSCRs. Consequently, information about gender, ethnicity, age and other demographic data were missing from the data extraction. However, the psychometric analysis reported in this paper did not require these data, and further analysis using the data to better understand the QoL of care home residents has used the complete DACHA MDS,^48^ in which demographic data has been populated through linkage with NHS data.^48 90^ The linked data has been compared with the overall care home resident population in England to explore representativeness: findings indicate that the DACHA MDS sample is comparable by sex and type of care home but the very old and ‘White’ ethnic group are over-represented.^86^

For DSCR data to be consistently used to populate a care home MDS, greater standardisation of the approach to missing data should be considered. Nonetheless, the evidence reported here indicates that it is feasible to routinely capture data about residents’ QoL through staff-proxies. The study has demonstrated that it is not feasible to consistently collect data from care home residents through self-report alone. Most residents will require help in the form of reading the questions, talking through the responses and marking the answers. A substantial proportion would be excluded entirely without using proxy-report. Three of the four QoL measures piloted had good psychometric properties for internal consistency and construct validity by hypothesis testing: the EQ-5D-5L (health-related QoL), the ASCOT-Proxy-Resident (social care-related QoL) and the ICECAP-O (capability well-being). As a key purpose of measuring resident QoL is to assess care quality and effectiveness; it is vital that the QoL measures included in the MDS are responsive to the quality, safety and effectiveness of care. This should be explored in future research.

This study is the first to pilot the inclusion of QoL measures in DSCRs in England. It was not possible to make specific recommendations about which of the three QoL measures with satisfactory performance should be prioritised for inclusion in an MDS. Each measures a different QoL construct, and, as such, further work would be required with key stakeholders, if a choice was required. There may be a strong case for including more than one, given their measurement of distinct constructs. The staff were not given training or detailed guidance beforehand, only the written instructions already included by the authors of the scales. Despite this, most measures were completed in full once staff made a start. The ASCOT-Proxy-Resident had slightly higher levels of missing data for some items (eg, dignity). This may also indicate that the staff would benefit from more guidance or support to interpret and complete these items as part of routine care. Ongoing work to support the use of ASCOT in care planning in care homes in Sweden^87^ indicates that these issues can be addressed by training key members of staff to be QoL champions, mentoring other staff. The care planning approach, which involves conversations with residents and family members, also better integrates QoL into routine care by identifying how practice will maintain or improve QoL. This is one of the core principles of the DACHA MDS^14^ and may be useful when considering the implementation of QoL measures in DSCRs in the future.

## Data Availability

Data are available upon reasonable request.
