# Supplementary material for: Assessing the feasibility of measuring residents’ quality of life in English care homes and the construct validity and internal consistency of measures completed by staff proxy: a cross-sectional study
Source: BMJ Open. 2025 Jan 29;15(1):e090684. doi: 10.1136/bmjopen-2024-090684 (PMC11781126; doi:10.1136/bmjopen-2024-090684)
Supplement: Supplementary file 1 [file bmjopen-2024-090684.reviewer_comments.pdf]

PEER REVIEW HISTORY

BMJ Open publishes all reviews undertaken for accepted manuscripts. Reviewers are asked to complete a checklist review form and are provided with free text boxes to elaborate on their assessment. These free text comments are reproduced below.

ARTICLE DETAILS

Title (Provisional)

Cross-sectional study assessing the feasibility of measuring residents Quality of Life in English care homes and assessing the construct validity and internal consistency of measures completed by staff-proxy.

Authors

Towers, Ann-Marie; Rand, Stacey; Allan, Stephen; Webster, Lucy Anne; Palmer, Sinead; Carroll, Rachael; Gordon, Adam L; Akdur, Gizdem; Smith, Nick; Burton, Jennifer; Killett, Anne; Hanratty, Barbara; Meyer, Julienne; Spilsbury, Karen; Goodman, Claire

---

VERSION 1 - REVIEW

**Reviewer**	**1**
**Name**	**Milte, Rachel**
**Affiliation**	**Flinders University, Rehabilitation, Aged and Extended Care**
**Date**	**16-Sep-2024**
**COI**	**Nil to declare**

Thank you for the opportunity to review this well written manuscript on a very important but previously under-researched topic - namely how best to measure quality of care from the perspective of the older people in care homes, where cognitive and communication difficults can hamper efforts to gain this information from the person themselves. I find the project has been clearly designed, with clear aims and objectives, and is clearly well written. I have only some minor comments, suggestions prior to publication.

couple of sentences about care homes - what they are and the terms used in other countries for similar services. Terminology changes from country to country, so this would just help orientate people from outside of the UK to the context of your research.

In the methods section, I was unclear about whether the data was cross sectional (i.e. one time point only) or whether this data was collected at multiple time points for the same individuals for inclusion?

In terms of the results, I found there was some comparisons that I thought would better fit in discussion rather than results. For example, in term s of the flood and ceiling effects you report comparison with community dwelling people in england, and in terms of compared with dutch and german translations of the ICECAP-O. I would usually expect this type of discussion in the discussion section.

The reporting of the contruct validity section in the results is very short. Please provide more detail (e.g. any differences across the different instruments or any domains of the instruments compared to others?)

A more general comment:

There is ongoing debate about whether to use external informants (e.g. family members) or employed staff for reporting quality of care. While it is true that staff reports on pain are commonly used, there is something different about asking staff to rate the quality of the care they provide, rather than performing assessment for clinical care purposes, not least because they would likely have received formal training on how to perform assessment for clinical care during formal training. More detailed consideration of the differences between assessment for care processes vs quality assessment by staff would be useful e.g. need for training or guidelines on how to assess? recommendations for analysts on how to incorporate assessments by staff into analysis? how to measure/ensure validity of responses and reduce bias? There may not be clear answers on how to answer all these questions now, but these are all worthy areas for future research I feel.

Reviewer 2

Name Engel, Lidia

Affiliation Monash University

Date 20-Sep-2024

COI I have previously published with Dr Stacey Rand who is listed as a co-author in this manuscript. However, this has not influenced my review of this paper.

Thank you for the opportunity to review this insightful paper. The measurement of quality of life in residential aged care is a critical area of research, and this study offers valuable findings. However, due to the pilot nature of the study and its design limitations, the results should be interpreted with caution. Below, I provide several suggestions for the authors.

• The DACHA study opted for proxy reporting by staff, a method that presents several challenges, particularly regarding the potential for bias. Could the authors elaborate on the rationale behind this decision? Relying solely on staff proxy reporting appears risky without addressing the extent of bias involved and its implications for interpreting the results.

• Page 9: Could the authors provide more detail on the consent process? Who was responsible for obtaining consent? How was the information communicated to the residents? Additionally, how did the team ensure informed consent for residents with limited capacity?

• Could the authors clarify which proxy perspective was adapted for the ICEACP-O measure?

• Who undertook the cognitive performance assessment and when?

• The data collection process lacks clarity and would benefit from further explanation. For instance, which staff members conducted the assessments? Were they provided with training? At what time of day were the assessments performed? Did the staff speak with the residents on the day of assessment to gauge their quality of life? This is especially important for instruments like the EQ-5D-5L, which uses a recall period of 'TODAY'.

• Does the MDS record the completion time? This would be very useful to report in order to understand the associated burden of the respective instrument?

• Why was forced completion required by provider 1 but not by provider 2? A clearer explanation of the data collection process would be helpful, including the training and instructions given to the providers.

• Could the authors also clarify how the providers were selected? Was there a reimbursement system in place for them?

• It seems that the authors used cross-sectional data but this is not very clear in the methods and should be described more clearly. Could the authors also describe why longitudinal data was not available? What is the purpose of the MDS for the future?

• It is unclear why the ASCOT proxy-proxy data is not included in Table 2, despite being collected. Since it is a relatively new measure, it would also be helpful to know which perspective led to more missing data.

• Page 12, line 12: The authors mention that provider 2 may have had more missing values due to residents passing away or being hospitalized. It would be helpful to know how many residents consented, how many were approached for data collection, and how many had missing data. Additionally, it would be useful to differentiate between complete missingness and partial missingness (i.e., where some items are missing).

• Table 4: There is a significant ceiling effect for QUALIDEM 3 (enjoyment of meals). Since the assessments were conducted by staff, could this have introduced bias regarding aspects of quality of life that are more closely linked to quality of care? The same concern applies to the ASCOT, which includes questions about food and cleanliness and also shows ceiling effects compared to health and wellbeing measures like the ICECAP-O or EQ-5D-5L. Could the authors discuss the implications of this for quality assessment?

• Page 13, line 43: The authors find that the ASCOT dignity item resulted in missing values possibly due to difficulties of understanding. The authors could cite literature showing that

also residents themselves find this item challenging (not only staff). See Engel et al. Patient. 2023 Nov;16(6):655-666.

• Page 14, line 3: The authors conclude that, due to mixed evidence for structural validity and internal consistency, they cannot recommend QUALIDEM for use in UK care homes. However, this conclusion seems overly strong, considering that the construct validity was adequate, which may be more significant than structural validity and internal consistency. Additionally, other psychometric properties should be examined before reaching such a conclusion. The authors also appear contradictory when they state in the following paragraph that all three measures have strong psychometric evidence.

• Page 14, line 28: The authors note that only a small proportion of residents were able to complete the single-item question independently, leading them to conclude that residents should not answer quality of life questions themselves due to the resulting missing values. I would caution against such a definitive conclusion. Firstly, it is possible that staff did not make sufficient attempts to gather this information from residents, as they had the option to proxy report, which was more convenient for them. Secondly, literature indicates a low level of agreement between staff and residents, suggesting that quality of life data collected via staff proxy reporting may not accurately reflect residents' true status. This should be carefully considered and balanced in the decision-making process.

• Could the authors address the policy landscape in Australia, which mandates self-reporting as the primary assessment method, followed by family proxy reporting, and staff proxy reporting only when no family member is available? Additionally, it would be beneficial for the authors to mention in the introduction the mandatory quality of life measurement in residential aged care facilities in Australia using the QOL-ACC measure.

Minor comments:

• Page 5, line 23: Could the authors add the corresponding reference for the statement that other countries have introduced MDS?

• Table 1: It's not clear what the values in brackets indicate

---

VERSION 1 - AUTHOR RESPONSE

Response to Reviewers' Comments

Reviewer 1

Thank you to Dr Milte for taking time to carefully review this submission. We are grateful for the positive feedback and suggestions on how to improve the paper.

1. Couple of sentences about care homes - what they are and the terms used in other countries for similar services. Terminology changes from country to country, so this would just help orientate people from outside of the UK to the context of your research.

RESPONSE: We have added an explanation at the start of the introduction. Specifically, we have defined broadly what care homes do in England and then distinguished between nursing and non-nursing homes to help orient an international audience.

2. In the methods section, I was unclear about whether the data was cross sectional (i.e. one time point only) or whether this data was collected at multiple time points for the same individuals for inclusion?

RESPONSE: We think Dr Milte may have missed this information, as it is already reported. The title says 'cross-sectional' and under Study Design it says: "This analysis draws on cross-sectional data from residents' DSCRs collected in wave one of the DACHA Study, which was a mixed-methods pilot of a prototype MDS (see study protocol for full details [1])."

3. In terms of the results, I found there was some comparisons that I thought would better fit in discussion rather than results. For example, in terms of the floor and ceiling effects you report comparison with community dwelling people in england, and in terms of compared with dutch and german translations of the ICECAP-O. I would usually expect this type of discussion in the discussion section.

RESPONSE: We have moved references to other work to the Discussion section and also added some text drawing out the wider points you have made below about staff ratings, care quality indicators and training/support for staff.

4. The reporting of the construct validity section in the results is very short. Please provide more detail (e.g. any differences across the different instruments or any domains of the instruments compared to others?)

RESPONSE: We had kept this concise because of concerns over word count. We have now provided more detail and have made more explicit the difference between the HRQoL measure and the other measures, including why EQ-5D-5L was only weakly associated with the over QoL item.

A more general comment:

There is ongoing debate about whether to use external informants (e.g. family members) or employed staff for reporting quality of care. While it is true that staff reports on pain are commonly used, there is something different about asking staff to rate the quality of the care they provide, rather than performing assessment for clinical care purposes, not least because they would likely have received formal training on how to perform assessment for clinical care during formal training. More detailed consideration of the differences between assessment for care processes vs quality assessment by staff would be useful e.g. need for training or guidelines on how to assess? recommendations for analysts on how to incorporate assessments by staff into analysis? how to measure/ensure validity of responses and reduce bias? There may not be clear answers on how to answer all these questions now, but these are all worthy areas for future research I feel.

RESPONSE: We have now added some text around these issues to the Discussion, with reference to the results we found and differences between ASCOT and the other measures.

Reviewer 2

Thank you to Dr Engel for taking time to carefully review this submission. We are grateful for the feedback and suggestions on how to improve the paper.

1) The DACHA study opted for proxy reporting by staff, a method that presents several challenges, particularly regarding the potential for bias. Could the authors elaborate on the rationale behind this decision? Relying solely on staff proxy reporting appears risky without addressing the extent of bias involved and its implications for interpreting the results.

RESPONSE: We understand that this is an important discussion point and that is why we dedicated three paragraphs in the introduction explaining why we chose this approach (starting the para which opens "The mode of completion (self-complete/proxy) is critical...").

We have cited several previous studies demonstrating that self-report is simply not feasible or possible with this population in England (something that is then supported by our own data in this paper – see, results for the QoL single item). We discuss the controversial nature of staff acting as proxies and refer to the wider literature about this. We argue that mixed-methods (which the lead author and her team have previously strongly advocated for and indeed developed tools for) are preferable but not feasible (referencing ASCOT work and Dementia Care Mapping work in this area) and note the inequity of relying on family as proxies (not everyone has an appropriate family member or friend). We then go on to reference the process we underwent to consult care home stakeholders about the tools included and features that were important (citation updated as this paper has been now been peer reviewed and published: Akdur G et al. National stakeholder consultation on how to measure care home residents' quality of life. [published online ahead of print September 25 2024]. Health Soc Care Deliv Res 2024. https://doi.org/10.3310/NPYT7562).

Recognising concerns about staff proxy report, we also included a single question on general QoL for residents to self-report alone or with help – being a single item question, we hypothesised that this might be more accessible for residents to complete than a standardised scale. However, even then less that 15% could complete without help from staff (see, Results).

We argue that proxy-report is not synonymous with self-report and should not be treated as such. Rather, it is another form of reporting which enables complete information on all residents, regardless of their mental capacity, family circumstances or health. The DACHA MDS worked with software vendors to integrate the measures into care home digital care records. These records are designed to be completed by staff and the advantage of this for an MDS in England is that all extracted data is comparable between and within services.

This paper sought to pilot the measures for the first time in DSCRs for the purposes of populating a care home MDS and evaluated the feasibility and psychometric properties of the measures in this context.

2) Page 9: Could the authors provide more detail on the consent process? Who was responsible for obtaining consent? How was the information communicated to the residents? Additionally, how did the team ensure informed consent for residents with limited capacity?

RESPONSE: we have added more detail here.

3) Could the authors clarify which proxy perspective was adapted for the ICEACP-O measure?

RESPONSE: Under Measures: ICECAP-O we have already noted: “The measure has not been specifically designed or adapted for proxy report. However, it has been applied in the context of older adult care homes as a proxy-report instrument with the recommendation (pending further evidence) that it ought to be completed by professional staff, rather than family members [54].”

The ICECAP-O does not have a version designed for proxy completion, therefore we simply asked staff to complete it on the residents’ behalf. We have added this as a limitation and topic of future research in the Discussion.

4) Who undertook the cognitive performance assessment and when?

RESPONSE: the MDS Cognitive performance Scale was added to the DSCR software like all the other measures and completed by staff. All data collection was complete during the

same period (see, Data Collection). We have added a sentence to clarify this under the measure's description.

5) The data collection process lacks clarity and would benefit from further explanation. For instance, which staff members conducted the assessments? Were they provided with training? At what time of day were the assessments performed? Did the staff speak with the residents on the day of assessment to gauge their quality of life? This is especially important for instruments like the EQ-5D-5L, which uses a recall period of 'TODAY'.

RESPONSE: we have added some additional detail under data collection about the general approach and specific details for each measure under 'Measures'.

6) Does the MDS record the completion time? This would be very useful to report in order to understand the associated burden of the respective instrument?

RESPONSE: This data was collected as part of the home's digital care records, being used as part of routine care, rather than as part of a separate MDS system. We therefore did not assess this information. As well as reporting missingness in this paper, we discuss burden in a qualitative paper exploring the perspectives of staff who completed the measures. See: Carroll R, Smith N, Palmer S, et al. Piloting a Minimum Data Set (MDS) in English care homes: a qualitative focus group and interview study of professional perspectives on implementation and use. Res Sq Published Online First: 2024. doi:https://doi.org/10.21203/rs.3.rs-4564243/v1)

7) Why was forced completion required by provider 1 but not by provider 2? A clearer explanation of the data collection process would be helpful, including the training and instructions given to the providers.

RESPONSE: In England digital care record systems are still relatively new and we were working with two different software vendors who had different requirements for measures within their systems. One of the recommendations of our work is that there needs to be greater standardisation across providers and we are informing central government policy around this. We have acknowledged that this is a limitation when discussing Feasibility in the Results.

8) Could the authors also clarify how the providers were selected? Was there a reimbursement system in place for them?

Software companies volunteered to take part in the research. The two that took part both had ‘cloud based’ systems (to facilitate data extraction). We have now added additional information to the relevant section, under Participants.

9) It seems that the authors used cross-sectional data but this is not very clear in the methods and should be described more clearly. Could the authors also describe why longitudinal data was not available? What is the purpose of the MDS for the future?

RESPONSE: Apologies if this wasn’t clear. We have reported this information under the headings recommended by COSMIN.

Study design (under Methods and Analysis)

This analysis draws on cross-sectional data from residents’ DSCRs collected in wave one of the DACHA Study, which was a mixed-methods pilot of a prototype MDS (see study protocol for full details [1]).

We have also reiterated this under ‘Data Collection’. This analysis was conducted only on the DSCR from wave one because the sample size was large, and we did not require longitudinal data to run the analysis presented here.

This paper (Title: Cross-sectional study assessing the feasibility of measuring residents’ Quality of Life in English care homes and assessing the construct validity and internal consistency of measures completed by staff-proxy) is not about the wider MDS. It focuses on the important issue of how resident QoL can be captured in digital care records. This was part of a larger study piloting an MDS for care homes for older adults in England. This work is reported here, along with a wider discussion about how a linked health and social care MDS might be used:

Gordon AL, Rand S, Crellin E, et al. Piloting a minimum data set for older people living in care homes in England: a developmental study. medRxiv 2024;2024.06.07. doi:https://doi.org/10.1101/2024.06.07.24308589 – currently addressing peer review comments for Age and Ageing.

10) It is unclear why the ASCOT proxy-proxy data is not included in Table 2, despite being collected. Since it is a relatively new measure, it would also be helpful to know which perspective led to more missing data.

We had previously not included it because:

"Two recent studies of the ASCOT-Proxy completed by family carers of people with dementia, living at home, and also with care home residents using the DACHA study sample, have found that of the two measures, only the ASCOT-Proxy-Person/Resident has the same structural characteristics as the original ASCOT self-completion version (SCT4) from which the ASCOT-Proxy was adapted [53,54]. From these studies, it was concluded that the ASCOT-Proxy-Proxy perspective is still useful to include, as it may improve the instrument's acceptability and face validity to proxy respondents, giving them an opportunity to express their own views as well as what they think the person feels [41]. However, the findings indicate that the ASCOT-Proxy-Resident should be the focus of future analyses, which is why we present only the ASCOT-Proxy-Resident here."

However, we appreciate that it is interesting for readers to be able to compare, especially as this is the first time we have asked staff to complete these measures in digital care records, therefore we have added this data to Table 2 and Table 3 for completeness. We have also added to the end of the paragraph above, "... with the exception of % missing data (since not reported elsewhere)."

11) Page 12, line 12: The authors mention that provider 2 may have had more missing values due to residents passing away or being hospitalized. It would be helpful to know how many residents consented, how many were approached for data collection, and how many had missing data. Additionally, it would be useful to differentiate between complete missingness and partial missingness (i.e., where some items are missing).

RESPONSE: Detailed information on resident recruitment and flow through the DACHA study is reported in the paper describing the main MDS pilot (Gordon et al, 2024 – noted above). We have now cited this paper and edited the text accordingly (under, Participants). We have indicated possible reasons why Provider 2 homes had more complete missing data but added a sentence noting a limitation of using the digital care record data only (without the linked health data) is that we cannot identify and exclude residents for whom measures were not completed due to death/hospitalisation.

We have revised Table 2, as well as the description in the Methods and Results, to refer to and report complete missingness (i.e. no items completed in the scale) compared to partial missingness (i.e. where only some items are missing). The columns on overall missingness are now redundant (as we have reported the breakdown) and have been deleted.

12) Table 4: There is a significant ceiling effect for QUALIDEM 3 (enjoyment of meals). Since the assessments were conducted by staff, could this have introduced bias regarding aspects of quality of life that are more closely linked to quality of care? The same concern applies to

the ASCOT, which includes questions about food and cleanliness and also shows ceiling effects compared to health and wellbeing measures like the ICECAP-O or EQ-5D-5L. Could the authors discuss the implications of this for quality assessment?

RESPONSE: We have now added a more detailed discussion of the ceiling effect and mean scores for ASCOT and discussed why measures that capture the impact of care are more likely to have ceiling effects in this context (See Discussion), especially compared to EQ5D and ICECAP-O, which have not been associated with care quality.

13) Page 13, line 43: The authors find that the ASCOT dignity item resulted in missing values possibly due to difficulties of understanding. The authors could cite literature showing that also residents themselves find this item challenging (not only staff).

RESPONSE: Thank you for this suggestion but, as outlined above, in England we do not expect residents to self-report due to high levels of cognitive impairment and dementia. This is one of the reasons we developed the mixed-methods toolkit for use in care homes. As such, we do not feel it is appropriate to refer to self-report here, as it may confound issues around capacity. We have cited proxy literature, which is comparable to the data collected here.

14) Page 14, line 3: The authors conclude that, due to mixed evidence for structural validity and internal consistency, they cannot recommend QUALIDEM for use in UK care homes. However, this conclusion seems overly strong, considering that the construct validity was adequate, which may be more significant than structural validity and internal consistency. Additionally, other psychometric properties should be examined before reaching such a conclusion. The authors also appear contradictory when they state in the following paragraph that all three measures have strong psychometric evidence.

RESPONSE: this paper was the first time that the English language translation of QUALIDEM had been psychometrically evaluated. Under COSMIN, a CFA would usually be run against the developer's subscales. Previous translations of QUALIDEM into Danish and German have found different subscales from the original Dutch language version, so we correspondingly ran and reported an EFA, and suggested alternative subscales for the English transaltion. This adds to the research evidence and provides a basis on which the English measure can be validated further but it also raises questions about the validity of the tool if each translation ends up with a different structure to the original version. As such, we are not able to recommend its use by the care home sector at this stage and have recommended further testing.

In the following paragraph we state: “Overall, the psychometric evidence (internal consistency, construct validity and also, structural validity, where appropriate) supported the use of the other three multi-item measures” – here we use the words ‘other three’ to refer to the ASCOT-Proxy, ICECAP-O and EQ5D.

15) Page 14, line 28: The authors note that only a small proportion of residents were able to complete the single-item question independently, leading them to conclude that residents should not answer quality of life questions themselves due to the resulting missing values. I would caution against such a definitive conclusion. Firstly, it is possible that staff did not make sufficient attempts to gather this information from residents, as they had the option to proxy report, which was more convenient for them. Secondly, literature indicates a low level of agreement between staff and residents, suggesting that quality of life data collected via staff proxy reporting may not accurately reflect residents’ true status. This should be carefully considered and balanced in the decision-making process.

RESPONSE: On the contrary, we would like to point the reviewer to the Results section. Of the n=613 cases where the single item ASCS QoL item was completed, 14.9% (n=91) were completed by the resident without help and 27.8% (n=170) were completed by staff proxy, without any involvement of the resident. The remaining responses, except one case of missing data, (57.3%, n=351) were completed by the resident with assistance from care staff, e.g. to read, talk through and/or translate questions. This indicates that staff did all they could to support residents to self-report, as only 27.8% were by proxy report. We do not conclude that this single item QoL question should not be included in an MDS, only that this provides supporting evidence that it is not feasible to expect residents to self-report using the longer, multi-item scales and that if we did rely on self-report, we would have high levels of missing data:

“It is likely that if residents had self-completed the longer QoL measures in DACHA, we would have had substantial missing data, affecting the ability to generate overall scores and interpret the results.”

As outlined above, we do not consider self-report and proxy-report to be equivalent and this is why it is important to understand whose view you are collecting – data from proxies should not be merged with data from residents themselves. Staff-proxy data will provide comparable information for all residents, not only those who can self-report and those who have family. Additional data could still be collected through family experience surveys and care planning activities.

(15) Could the authors address the policy landscape in Australia, which mandates self-reporting as the primary assessment method, followed by family proxy reporting, and staff proxy reporting only when no family member is available? Additionally, it would be beneficial for the authors to mention in the introduction the mandatory quality of life measurement in residential aged care facilities in Australia using the QOL-ACC measure.

RESPONSE: We have added a reference to QoL being mandatory in Australia to the introduction and also added this when discussion mode of completion of QoL measures in care homes for older people (see, introduction section):

“The mode of completion (self-complete/proxy) is critical. In Australia, where QoL measurement is mandated in residential aged care, self-report is the primary assessment method and indeed data reported for the first quarter of 2024 indicates that 85% of residents were able to self-report via survey (30%) or facilitated interview (55%) (REF). However, in England, response rates to QoL measures amongst care home residents are very low, with relatively few residents able to self-report [4,31–33] highlighting a significant methodological challenge regarding the routine measurement of QoL for this population.”

(16) Minor comments:

• Page 5, line 23: Could the authors add the corresponding reference for the statement that other countries have introduced MDS?

We have added:

de Almeida Mello J, Wellens NIH, Hermans K, De Stampa M, Cerase V, Vereker N, Jónsson PV, Finne-Soveri H, Meehan B, Declercq A. The Implementation of Integrated Health Information Systems – Research Studies from 7 Countries Involving the InterRAI Assessment System. International Journal of Integrated Care, 2023; 23(1): 8, 1–24. DOI: https://doi.org/10.5334/ijic.6968

• Table 1: It’s not clear what the values in brackets indicate

Some factors loaded onto more than one factor, in which case items were attributed to the highest loading factor with the secondary factor still reported in brackets. We have added this to the text body and table footnote.

VERSION 2 - REVIEW

**Reviewer**	**2**
**Name**	**Engel, Lidia**
**Affiliation**	**Monash University**
**Date**	**25-Nov-2024**
**COI**	

The authors have sufficiently addressed my comments. My only last suggestion is to include the ASCOT proxy-proxy results also in Table 4.

VERSION 2 - AUTHOR RESPONSE

We are grateful to Dr Engel for approving the revisions made. Regarding the further suggestion of adding the ASCOT proxy-proxy results to Table 4, we have already explained in the text why we have not done this and we think it may be unhelpful. Please see note under Table 4:

The descriptive statistics and psychometrics are only reported further for the ASCOT-Proxy-Resident, using preference weights developed for the ASCOT-SCT4 (Netten et al, 2012). For further discussion and justification of our focus on ASCOT-Proxy-Resident, not –Proxy-Proxy, see (Rand et al, 2024; Silarova et al, 2023).

We also discuss this in the article:

“However, the findings indicate that the ASCOT-Proxy-Resident should be the focus of future analyses, which is why we present only the ASCOT-Proxy-Resident here, with the exception of % missing data (since not reported elsewhere).”
